# Postoperative Pain Following Single-Visit Nonsurgical Retreatment Using Minimally Invasive Rotary vs. Reciprocating Nickel-Titanium File Systems: A Two-Arm Parallel Randomized Clinical Trial

**DOI:** 10.1155/prm/6826535

**Published:** 2025-09-08

**Authors:** Hüseyin Gürkan Güneç, Büşra Pehlivan, Celalettin Topbaş, Abdurrahman Kerim Kul, Dursun Ali Şirin

**Affiliations:** Department of Endodontics, Hamidiye Faculty of Dentistry, University of Health Sciences, İstanbul, Türkiye

**Keywords:** apical periodontitis, minimally invasive files, postoperative pain, retreatment, single visit treatment

## Abstract

**Objective:** This study aimed to compare postoperative pain following the single-visit retreatment procedures of asymptomatic and symptomatic teeth using two different nickel-titanium file systems.

**Materials and Methods:** Eighty patients were divided into symptomatic and asymptomatic groups, each further subdivided based on the use of rotary or reciprocating files. Retreatment involved removal of filling material with one flare and MicroMega REMOVER files, followed by shaping with one curve mini (rotary) or One RECI (reciprocating) files. Postoperative pain was recorded using a visual analogue scale (VAS) at 24 h, 48 h, 72 h, 7 days, and 14 days. Data were analyzed using Shapiro–Wilk, Mann–Whitney *U*, Kruskal–Wallis, Dunn–Bonferroni, and Pearson chi-square tests (*p* < 0.05).

**Results:** No significant differences in postoperative pain were found among the four groups. Pain levels were not associated with sex, age, or tooth position. Analgesic use significantly decreased over time in all groups except the asymptomatic rotary group. Patients requiring analgesics reported higher pain scores within the first 48 h (*p* < 0.05).

**Conclusions:** Postoperative pain was low and comparable across all groups. File kinematics (rotary vs. reciprocating) did not influence pain outcomes. Single-visit retreatment is a viable alternative to multivisit procedures for both symptomatic and asymptomatic cases.

**Clinical Relevance:** This study supports the clinical feasibility of single-visit root canal retreatment, potentially improving patient comfort and reducing chair time.

**Trial Registration:** ClinicalTrials.gov identifier: NCT06357481

## 1. Introduction

Root canal retreatment (RCRT) refers to a procedure to regain healthy periapical tissues when the teeth have undergone root canal treatment (RCT) and are reinfected due to apical or coronal leakage or after an inadequate RCT [1–3]. When primary RCT fails, the first treatment option is nonsurgical RCRT to eliminate the infection [4]. In general, the reported success rate of RCRT ranges from 62% to 91% [5, 6].

The apical region of a tooth with a periapical radiolucency may not always be symptomatic (ST) [7]. However, the clustering of some specific microorganisms, presumed to have increased virulence, causes a chronically inflamed tooth with periapical radiolucency to show symptoms. Radiolucency detected on radiography in clinically asymptomatic (AT) teeth with periapical lesions does not always indicate failure; it can mean that the lesion size is decreasing or has not fully healed [8, 9]. In such circumstances, further follow-up time may be needed to determine whether the treatment is successful.

Before RCRT, the average incidence of successfully treated clinically ST teeth is 74.2%; the success rate is 85.7% for AT teeth and 88.9% for fistula-detected teeth. Successful RCT requires a healing or healed lesion in the periapical region, with hermetic root canal filling on radiographs and no clinical symptoms of pain [10].

After retreatment of previously root canal-treated teeth, postoperative pain may occur. The response to pain varies depending on the situation, and the perception of pain is not always continuous. Even though endodontic instruments do not protrude into the periradicular tissues during the RCRT procedure, debris, root canal filling materials, irrigants, remaining pulp tissue, and microorganisms tend to do so [11]. Correlations have thus been found between apical extrusion and periradicular inflammation, postoperative pain, flare-ups, and delayed periapical healing [12–14].

The cutting and shaping effectiveness of the files is directly impacted by the design variations, flexibility, and deformation of the file systems. Consequently, the amount of debris extruded apically may also vary [15–17]. Minimally invasive endodontics (MIE) is an approach that aims to minimize structural changes after treatment. It includes the treatment and prevention of pulp diseases and apical periodontitis, as well as optimal root canal dentine preservation. The MIE method is safe, accurate, and time-saving. The risk of apical debris extrusion in the operation of minimally invasive files is minimal [18]. File systems that cause less pain after retreatment are preferred because they decrease the risk of flare-ups by minimizing the amount of apically extruded dentin [15, 19]. This intervention is expected to result in a considerable decrease in postoperative pain.

The primary goal of this randomized clinical study was to evaluate postoperative pain following single-visit RCRT for AT and ST mandibular premolars using minimally invasive reciprocating and rotary file systems. It aimed to compare the effects of the one curve mini (OCM) and one RECI (OR) nickel-titanium file systems on postoperative pain after nonsurgical endodontic retreatment of ST and AT teeth. Our null hypothesis is that none of the RCRT protocols would significantly affect the postoperative pain levels in a single-visit treatment.

## 2. Materials and Methods

A prospective, single-center, single-visit, randomized clinical trial was designed, with the protocol approved by the local clinical research ethics committee of Health Sciences University Hamidiye Clinical Human Research (decision no: SBU-2023/1/23-2). We confirmed that all research was performed in accordance with relevant guidelines and regulations and included in our manuscript a statement confirming that informed consent was obtained from all participants. All patients invited to participate in this trial were informed of the procedure protocols, benefits, and risks. All participants read and signed a form giving their consent to participate, and they received a copy. The study protocol was recorded in https://www.ClinicalTrials.gov databases (10/04/2024). We used the CONSORT checklist when writing our report [20].

### 2.1. Sample Size Calculation

Prior to the study, the required minimum sample size was calculated using the G∗Power 3.1 software with the following criteria: “Power (1-beta) 0.80,” “alpha (type 1 error rate): 0.05” and effect size (f): 0.4 [21]. Based on the computation, it was concluded that a total sample size of 80, 20 per group, would be sufficient.

### 2.2. Patient Selection and Allocation

One hundred twenty-one adult patients (aged 18–67 years) with a noncontributory medical history presented for endodontic retreatment to the Department of Endodontics, Hamidiye Dental Faculty of Health Sciences University, between January 2023 and January 2024. Patients were excluded from the study if any of the following conditions were observed: age under 18 years, complicating systemic disease, allergies to local anesthetic agents, acute apical abscesses, anti-inflammatory or antibiotic intake in the 7 days before treatment, periodontal pockets deeper than 5 mm or more mobility than Level 1, open apex that had not completed root development, bruxism or teeth grinding, pregnancy, painkiller use in the 24 h before the procedure, presence of external root resorption, root fracture, root perforation, and calcified root canals [22]. After 41 patients were excluded, our study involved the retreatment of 80 patients with AT or ST lower premolar teeth. An independent investigator, blinded to the patients' treatment assignments, performed patient preoperative status assessments before the start of treatment and throughout the study period.

### 2.3. Analysis of Preoperative Pain

Pain was evaluated with a visual analog scale (VAS). Before the treatment, all patients were given a pain report form on which to indicate their preoperative level of pain. The clinician first completed an example with each patient to confirm they understood the instructions (0: no pain, 1-2: discomfort, 3-4: mild, 5: moderate, 6-7: severe, 8-9: very severe, 10: worst pain possible) [23].

### 2.4. Randomization and RCRT Procedure

**Figure 1:**
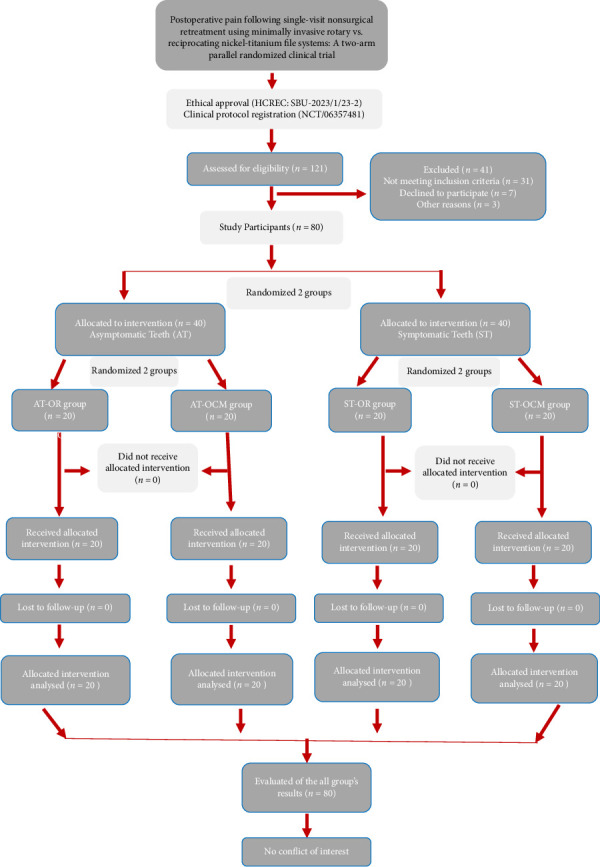
Flowchart of study participants during the inclusion, exclusion, and grouping processes.

Once it was confirmed that the patient met the inclusion criteria, the list was checked by another blinded investigator, who also evaluated the nonsurgical endodontic retreatment procedures to determine which group the patient would be assigned to either the AT group (preoperative VAS score; 0 or no pain) or the ST group (preoperative VAS score from discomfort to worst pain possible, 1-2–10). Before the operator started the retreatment procedures, a total of 80 patients were divided into four subgroups. (AT-OR, AT-OCM, ST-OR, and ST-OCM groups) (Figure 1). A randomization procedure (https://www.random.org) was used to randomly assign the patients to the rotary and reciprocating retreatment subgroups [20].

All treatment procedures were done by a single operator. Teeth were anesthetized using articaine hydrochloride (Primacaine, Pierre Rolland, Bordeaux, France) with epinephrine 1:200,000. Each tooth was isolated with a rubber dam. After the access cavity preparation and localization of the canal orifices old root canal filling materials were removed with the MicroMega one flare (OF) and REMOVER (RM) files (MicroMega, Besançon, France). The OF and RM files were used with full rotational motion (350 rpm speed and 2.5 N/cm torque) with the Dual Move (MicroMega, Besançon, France) endodontic motor. After the filling material was removed, the canals were irrigated with 5.25% NaOCl solution using an Accuject plastic irrigation needle tip (Dentsply Sirona, NY, USA). The removal of filling material was considered complete when no gutta-percha remains were observed under the dental operation microscopes (Labomed Magna, Culver City, CA, USA), and a periapical radiograph was taken for confirmation. Apical patency was controlled with a #10 K-file, and canal length was measured with a #10 or #15 K-file using a Morita Root Zx Mini apex locator.

**Figure 2:**
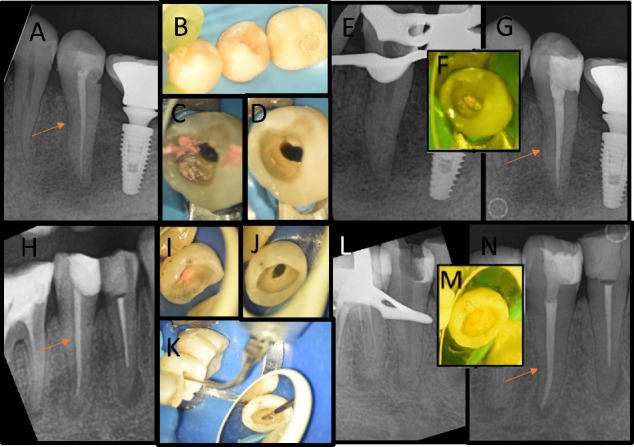
Case 1. ST-OCM group. (a) Lower second premolar tooth that had root canal treatment 3 years ago. The patient was diagnosed with symptomatic apical periodontitis. Periapical lesion and an unsuccessful restoration are present. (b) The image shows the intraoral condition of the tooth before the retreatment. (c) After the proximal walls have been made, the root canal filling was removed using MicroMega REMOVER, one flare, and one curve mini files. (d and e) The complete removal of the root canal filling was confirmed through the use of an operating microscope and periapical radiography. (f and g) Intraoral view and postoperative periapical radiography image after root canal filling was completed. Case 2. ST-OR group (h) lower second premolar tooth with symptomatic apical periodontitis, root canal treatment was performed 4 years ago. A periapical lesion is present. (i) Intraoral view after removal of the old coronal restoration. (j) Build up the proximal wall with composite resin. Checking using an operating microscope that the root canal filling has been completely removed with MicroMega REMOVER, one flare, and one RECI. (k) İrrigation activation with ultrasonic device. (l) The complete removal of the root canal filling was confirmed through the periapical radiography. (m and n) Intraoral view and postoperative periapical radiography image after root canal filling was completed.

The rotary and reciprocating file system used for the nonsurgical retreatments, comprised of the MicroMega OCM and OR systems, included 25/0.04, 25/0.06, 35/0.04, and 45/0.04 files to effectively remove the infected apical dentine. During the RCRT procedures, 5.25% NaOCl solution was used for irrigation after the use of each file. The final irrigation activation was applied to the canals three times per 20 s using the ultrasonic activation device (Newtron Booster; Satelec, Merignac, France) with 5 mL of 5.25% NaOCl, 17% EDTA, and 5.25% NaOCl, respectively. 5 mL of sterile saline was used to rinse the canals. The root canals were dried with paper points and filled with #45.04 gutta-percha cones (master cone compatible with the file system used) and an AH Plus (Dentsply Sirona, NY, USA) epoxy resin-based root canal sealer, using a lateral compaction technique. The access cavities were restored using a composite resin material with a direct adhesive technique during the same visit (Figure 2).

### 2.5. Analysis of Postoperative Pain

**Figure 3:**
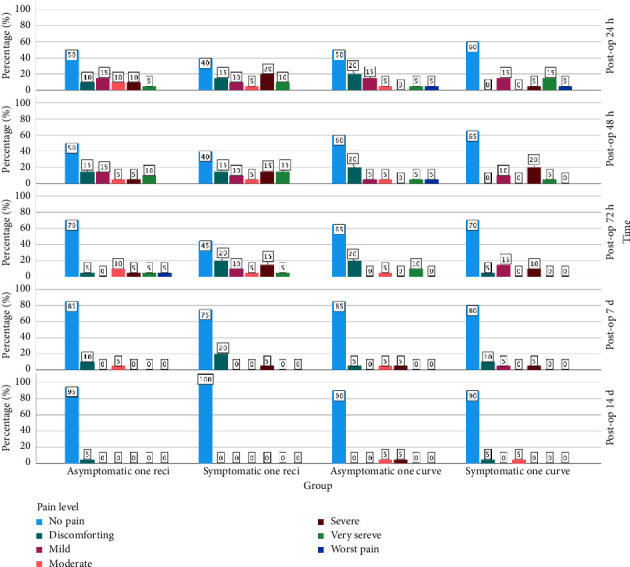
Percentage of postoperative pain levels between the different times.

After the endodontic retreatment procedure, all patients noted the pain level they felt from the first day to 14 days (24, 48, and 72 h and 7 and 14 days) on the VAS scale and gave the completed forms to the operator. Postoperative pain was ranked from 0 to 10 on a horizontal line and recorded daily, at the same time every day. Patients recorded when they felt pain (morning, evening, night, or all day) and whether they used analgesics (ibuprofen 600 mg; time and number taken), in which case they were asked to rate their pain before and after taking the analgesic. After the data were collected, statistical calculations and tables were used to evaluate whether statistically significant differences existed between the four groups (Figure 3).

### 2.6. Statistical Analysis

Descriptive statistics were calculated for each variable and expressed as either the mean and standard deviation or the median and range for continuous and ordinal variables. They were expressed as *n* and *n*% for categorical variables. Before hypothesis testing, the data were assessed for normality using the Shapiro–Wilk test and for homogeneity of variance using the Levene test, as required for parametric test assumptions. The Mann–Whitney *U* test was used for the comparison of two independent groups, whereas the Kruskal–Wallis test was employed for the comparison of more than two independent groups. The Friedman test was used to examine differences in measurements over time. A Dunn–Bonferroni test was applied as a post hoc procedure after significant differences were found. Pearson chi-square or Fisher–Freeman–Halton tests were used to examine the frequency distribution of categorical variables after considering the distribution of expected counts. Statistical analysis was performed using SPSS 21. The significance level was set at *p* < 0.05 for all statistical analyses.

## 3. Results

The data of 80 patients were thus analyzed. The demographic data and statistical comparisons are summarized in Table 1. No significant correlations existed between sex, tooth localization, age, and preoperative and postoperative pain at any time (*p* > 0.05).

The preoperative key pain vas score values were, respectively, 0, 4.6, 0, 4.8 (AT-OR, ST-OR, AT-OCM, ST-OCM) for each group (Table 2). The postoperative VAS score values were 2.25, 3.0, 2.1, 2.6 (24 h); 2.2, 2.95, 1.65, 2.0 (48 h); 1.8, 2.5, 1.35, 1.3 (72 h); 0.45, 0.60, 0.70, 0.65 (7^th^ day); 0.05, 0, 0.55, 0.3 (14^th^ day). No statistically significant difference was found between the groups in terms of pain scores obtained at each time point (*p* > 0.05). The highest levels of pain occurred during the first 24 h, followed by a significant reduction in all groups except the ST-OCM group, on Day 7. In the ST-OCM group, a significant decrease in pain was observed on Day 14, compared to the initial measurement. However, a statistically significant decrease in pain scores was observed for each group over time (*p* < 0.05; Table 3). No statistically significant difference was found in the frequency distributions of pain levels among the groups during any period (*p* > 0.05; Table 4).

For each group, average VAS in analgesic users and nonusers (AT-OR, ST-OR, AT-OCM, ST-OCM groups) were 5.8/1.06, 5.4/2.2, 7.0/0.87, 8.5/1.12 (24 h); 6.5/1.12, 7.16/1.14, 7.66/0.58, 7.0/0.81 (48 h); 7.75/0.43, 6.16/0.92, 7.33/0.29, 5.0/0.88 (72 h); 3.5/0.11, 3.5/0.27, 6.0/0.11, 4.0/0.47 (7^th^ day); 1.0/0, 0, 5.5/0, 0/0.3 (14^th^ day) for each group. Regardless of the study group in Table 5, patients who take an analgesic generally had different pain scores than the others for almost all time points.

## 4. Discussion

This prospective randomized clinical study aimed to assess the frequency of pain following single-visit endodontic retreatment in ST and AT teeth using rotary or reciprocating files. Single-visit RCT has become increasingly common in recent years. When compared to treatments completed in multivisits, single-visit treatments show better healing rates [24]. According to a Toronto study, single-visit retreatment showed better results than multiple visits for teeth with preoperative apical periodontitis [25]. Regarding the retreatment protocol, the results of recent clinical research evaluating the outcomes of single-visit RCRTs showed a high success rate [26]. After single-visit retreatments, 90.9% of the teeth in that study had healed, and 98.2% were still functional and AT. A systematic review of postoperative pain found no convincing evidence to support the claim that single-visit and multiple-visit RCT varied in terms of postoperative pain or flare-up [27]. Concerning endodontic retreatment specifically, Yoldas et al. discovered that two-visit endodontic retreatment with intracanal medication was successful in lowering postoperative pain for previously ST teeth and reducing the number of flare-ups in all cases [28]. However, even when performing single-visit endodontic retreatment, low pain rates were observed in the current study. Our study evaluated posttreatment pain conditions of ST and AT premolar teeth undergoing retreatment with a single visit.

We selected the single-root mandibular premolar tooth criterion for standardization in our study since postretreatment pain is multifactorial. We used two different file movements to carry out the retreatment procedure. Treatment predictability for short-term follow-up of postoperative pain can be achieved by using both systems to remove old root canal fillings and shaping root canals during endodontic retreatment. In patients who had nonsurgical retreatment, differences were observed in the pain scale used as a quantitative measure for statistical analysis (*p* < 0.05). Statistical differences between the instrument groups (reciprocating and rotary) were discovered following a multiple regression analysis of numerical pain values. Nonsurgical retreatment with reciprocating instruments produced postoperative pain that was less severe than that using rotary instrument [23]. Adequate apical preparation and purposeful enlargement of the apical foramen reportedly lower the microbial population, leading to better outcomes and reduced postoperative pain. However, the apical extrusion of debris containing dentine chips, necrotic pulp tissue, and bacteria and their by-products may result in postoperative pain [29]. The quantity of extruded debris and the pathogenicity of the microorganisms influence the degree of intensity of the inflammation [30]. By comparing reciprocating and rotary movements during RCRT, laboratory studies have found reduced debris extrusion, while other studies have found the opposite [27, 30, 31]. The distinction between rotary and reciprocating systems may be due to variations in motion kinematics and the number of instruments [27]. The results of these studies indicate that files with a reciprocating motion should result in less postoperative pain due to the decreased apical extrusion.

A new generation file called the RM undergoes electro-polishing and heat treatment as part of a developed thermomechanical process called C wire. The RM file's taper value and thermomechanical process may increase the success rate for single-root teeth before using rotary and reciprocating file systems [32]. In the final irrigation, an ultrasonic activation device was used to activate irrigation solutions to perform better canal disinfection [33]. Using a microscope is beneficial in overcoming challenging cases in endodontics [34]. For better visibility, a dental operating microscope was used in each of our procedures. We believe this may decrease postoperative pain by improving the success rate of retreatment.

Age, sex, and tooth location are considered important factors in the assessment of the VAS scale. The VAS demonstrates the importance of how postoperative pain is assessed and measured. Techniques for reporting patients' pain must be simple enough for both patients and investigators to understand. We selected the VAS for use in our study because it satisfies these requirements and has been utilized extensively in investigations [27, 35]. It was used to grade postoperative pain into categories of none, discomfort, mild, moderate, severe, extremely severe, and worst pain possible. The VAS is considered a valid and reliable measure of pain [36].

Clinical implications of postoperative pain rates after RCT vary among studies, with some reporting high rates [37, 38] and others reporting low rates [39, 40]. In a study comparing single-visit and multivisit treatment groups, participants in the single-visit group reported higher rates of pain within a week [41]. Although some studies have shown that intracanal medicament application significantly reduces postoperative pain, others have shown that single-visit RCT causes equally or even less postoperative pain than multivisit treatments [42]. Although opinions differ, the minimally invasive file systems and ultrasonic activation used in our study increased the advantages of single-visit treatment. So in our study, the patients who had AT and ST periodontitis began having different pain preoperatively and ended up with similar pain postoperatively because of using minimally invasive Ni-Ti files, which agrees with previous literature. Ultrasonic activation generates high-speed flow during irrigation, which helps to remove more debris and activates the irrigation fluids to reach the accessory canals more effectively, increasing treatment success. Importantly, many patients also fail to attend subsequent appointments after the initial pulp removal session due to pain relief, resulting in treatment failure. Retreatment was administered in a single visit in our study due to its practicality and higher patient acceptance rate. Our study's statistical data also support a significant decrease in pain conditions with a single visit.

Agreement with previous studies has explored the effects of single-visit RCT on postoperative pain in ST and AT teeth. In one study, single-visit endodontic treatments were applied to both ST and AT cases, resulting in successful outcomes [43]. Single-visit and multivisit endodontic treatments of ST and AT teeth demonstrated a similar incidence of postoperative complications [39, 44]. Whether a difference exists in the incidence of postoperative pain between ST and AT teeth that have undergone RCT has not been established. However, the decrease in the incidence of postoperative pain after RCT suggests that it may also decrease after retreatment. Although postoperative pain is expected to decrease over time, treatment success has been increased by using a minimally invasive file system to reduce the incidence of postoperative pain in single-visit treatment of ST and AT teeth [45]. Minimally invasive files are used to achieve success in RCT while preserving as much healthy coronal, cervical, and radicular tooth structure as possible. One study showed that using the SAF and Xp Shaper, a minimally invasive file, reduced postoperative pain by reducing the amount of shaping required [18]. Similarly, the minimally invasive file systems OCM and OR use heat-treatment technology by producing C-wire to perform minimally invasive shaping and are more compatible with canal anatomy [46]. Using these shaping systems, we observed a reduction in postoperative pain levels for each time point; this can comfort patients due to less postoperative pain and less need for analgesic taken. Furthermore, the reason for the significant decrease in pain in the following days in the OCM group may be that the pain tolerance of these patients was higher and the inflammatory response in the periapical region was suppressed more than in the other groups.

Notably, subjective assessment of pain is a fundamental issue in postoperative pain research and is the basis for decisions regarding analgesic needs. Because nonsteroidal anti-inflammatory drugs are recommended as the first-choice medication for postoperative pain treatment after endodontic procedures, ibuprofen was chosen. Our study and that of Comparin et al. found ibuprofen to be successful for pain relief following endodontic retreatment [22].

There is ongoing debate regarding the impact of rotary and reciprocating file systems on postoperative pain. Çanakçı et al. reported that reciprocating systems may lead to greater apical extrusion compared to rotary systems, potentially increasing postoperative discomfort [46]. In contrast, Uzunoglu et al. suggested that reciprocating systems result in less apical extrusion and may reduce postoperative pain [47]. This randomized clinical trial evaluated two different file systems in nonsurgical endodontic retreatment, focusing on the incidence, intensity, and duration of postoperative pain. Our findings showed no significant difference in pain outcomes between rotary and reciprocating files, in alignment with previous literature [23].

## 5. Limitations

The primary limitation of this study was the challenge of maintaining consistent clinical follow-up, compounded by the inherently subjective nature of pain assessment, even when using the VAS. Despite these challenges—common in postoperative pain research—efforts were made to ensure that patients completed the VAS based on accurate and objective guidance. Additionally, achieving the desired sample size within each group proved difficult due to the clinical setting. The study was also limited by being conducted at a single institution, with all retreatment procedures performed by the same endodontist, which may restrict the generalizability of the findings and limit the diversity of clinical perspectives.

## 6. Future Directions

These findings in single-visit endodontic retreatment highlight the need for deeper analysis in areas such as postoperative pain management, particularly comparing outcomes between general dentists and specialists. Future studies on postoperative pain could benefit from comparing minimally invasive techniques with conventional root canal procedures, focusing on a single endodontic condition to reduce variability. Additionally, treatments should be performed by experienced clinicians, with careful consideration of analgesic use and the risk of flare-ups, to ensure more accurate and reliable evaluation of these inherently subjective clinical outcomes.

## 7. Conclusion

To our knowledge, no previous studies have evaluated postoperative pain following the retreatment of ST and AT teeth using the OCM and OR file systems. In our study, no statistically significant differences were observed among the four groups after single-visit retreatment, thereby supporting the null hypothesis. The use of two minimally invasive nickel-titanium systems—one with continuous rotary motion and the other with reciprocating motion—resulted in comparable levels of postoperative pain and analgesic intake in both ST and AT single-rooted premolars. Additionally, no significant correlations were found between postoperative pain and variables such as sex, tooth position, age, or preoperative pain levels. The importance of this study was to ensure completing a single-visit treatment under ST or AT conditions as an alternative to multiple-visit treatments in nonsurgical retreatments.

## Figures and Tables

**Table 1:** Baseline demographic and clinical features of patients in study groups.

Variable	Subcategory	One RECI file	One curve mini file	*p* value^*x*^
		*n* (%)	*n* (%)	
Sex	Male	16 (50%)	16 (50%)	1
	Female	24 (50%)	24 (50%)	

Tooth	Mandibular left premolar	16 (43.2%)	21 (56.8%)	0.262
	Mandibular right premolar	24 (55.8%)	19 (44.2%)	

Clinical status	Absence of preoperative pain (asymptomatic)	20 (50%)	20 (50%)	1
	Presence of preoperative pain (symptomatic)	20 (50%)	20 (50%)	

Age group	< 30 years	11 (52.4%)	10 (47.6%)	0.963
	30–50 years	18 (48.6%)	19 (51.4%)	
	> 50 years	11 (50%)	11 (50%)	

^*x*^Pearson chi-square.

**Table 2:** Frequency and percentage of preoperative pain (*n* = 20 for each group).

Group	Pain levels						
	No pain	Discomforting	Mild	Moderate	Severe	Very severe	Worst pain
	*n* (%)	*n* (%)	*n* (%)	*n* (%)	*n* (%)	*n* (%)	*n* (%)
Asymptomatic one RECI	20 (100%)	0 (0%)	0 (0%)	0 (0%)	0 (0%)	0 (0%)	0 (0%)
Symptomatic one RECI	0 (0%)	7 (35%)	3 (15%)	5 (25%)	2 (10%)	1 (5%)	2 (10%)
Asymptomatic one curve	20 (100%)	0 (0%)	0 (0%)	0 (0%)	0 (0%)	0 (0%)	0 (0%)
Symptomatic one curve	0 (0%)	2 (10%)	7 (35%)	4 (20%)	6 (30%)	0 (0%)	1 (5%)

**Table 3:** Mean and median postoperative pain scores at each time point.

Group	24 h		48 h		72 h		Day 7		Day 14		*p* value^*x*^
	Mean ± SD	Median (range)	Mean ± SD	Median (range)	Mean ± SD	Median (range)	Mean ± SD	Median (range)	Mean ± SD	Median (range)	
Asymptomatic one RECI	2.25 ± 2.65	1 (8)^a^	2.2 ± 2.76	1 (8)^ab^	1.8 ± 3.16	0 (10)^abc^	0.5 ± 1.24	0 (5)^bc^	0.1 ± 0.33	0 (1)^c^	< 0.001
Symptomatic one RECI	3 ± 3.2	2 (9)^a^	2.95 ± 3.17	2 (8)^a^	2.5 ± 2.89	2 (8)^ab^	0.6 ± 1.6	0 (7)^bc^	0.1 ± 0.33	0 (1)^c^	< 0.001
Asymptomatic one curve mini	2.1 ± 3.01	0.5 (10)^a^	1.65 ± 2.87	0 (10)^ab^	1.35 ± 2.72	0 (9)^ab^	0.7 ± 1.89	0 (7)^b^	0.6 ± 1.7	0 (6)^b^	< 0.001
Symptomatic one curve mini	2.6 ± 3.68	0 (10)^a^	2.05 ± 3.09	0 (8)^ab^	1.3 ± 2.25	0 (7)^ab^	0.65 ± 1.6	0 (6)^ab^	0.35 ± 1.14	0 (5)^b^	< 0.001
*p* value^*y*^	0.799		0.465		0.356		0.902		0.497		

*Note:* ^a,b,c^Different letters in the same row represent a statistically significant difference between each time point.

^*x*^Freidman test.

^*y*^Kruskal–Wallis test.

**Table 4:** Frequency and percentage of postoperative pain.

Postoperative time	Group	Pain levels							*p* value^*x*^
		No pain	Discomforting	Mild	Moderate	Severe	Very severe	Worst pain	
		*n* (%)	*n* (%)	*n* (%)	*n* (%)	*n* (%)	*n* (%)	*n* (%)	
24 h	Asymptomatic one RECI	10 (50%)	2 (10%)	3 (15%)	2 (10%)	2 (10%)	1 (5%)	0 (0%)	0.665
	Symptomatic one RECI	8 (40%)	3 (15%)	2 (10%)	1 (5%)	4 (20%)	2 (10%)	0 (0%)	
	Asymptomatic one curve	10 (50%)	4 (20%)	3 (15%)	1 (5%)	0 (0%)	1 (5%)	1 (5%)	
	Symptomatic one curve	12 (60%)	0 (0%)	3 (15%)	0 (0%)	1 (5%)	3 (15%)	1 (5%)	

48 h	Asymptomatic one RECI	10 (50%)	3 (15%)	3 (15%)	1 (5%)	1 (5%)	2 (10%)	0 (0%)	0.594
	Symptomatic one RECI	8 (40%)	3 (15%)	2 (10%)	1 (5%)	3 (15%)	3 (15%)	0 (0%)	
	Asymptomatic one curve	12 (60%)	4 (20%)	1 (5%)	1 (5%)	0 (0%)	1 (5%)	1 (5%)	
	Symptomatic one curve	13 (65%)	0 (0%)	2 (10%)	0 (0%)	4 (20%)	1 (5%)	0 (0%)	

72 h	Asymptomatic one RECI	14 (70%)	1 (5%)	0 (0%)	2 (10%)	1 (5%)	1 (5%)	1 (5%)	0.282
	Symptomatic one RECI	9 (45%)	4 (20%)	2 (10%)	1 (5%)	3 (15%)	1 (5%)	0 (0%)	
	Asymptomatic one curve	13 (65%)	4 (20%)	0 (0%)	1 (5%)	0 (0%)	2 (10%)	0 (0%)	
	Symptomatic one curve	14 (70%)	1 (5%)	3 (15%)	0 (0%)	2 (10%)	0 (0%)	0 (0%)	

Day 7	Asymptomatic one RECI	17 (85%)	2 (10%)	0 (0%)	1 (5%)	0 (0%)	0 (0%)	0 (0%)	0.881
	Symptomatic one RECI	15 (75%)	4 (20%)	0 (0%)	0 (0%)	1 (5%)	0 (0%)	0 (0%)	
	Asymptomatic one curve	17 (85%)	1 (5%)	0 (0%)	1 (5%)	1 (5%)	0 (0%)	0 (0%)	
	Symptomatic one curve	16 (80%)	2 (10%)	1 (5%)	0 (0%)	1 (5%)	0 (0%)	0 (0%)	

Day 14	Asymptomatic one RECI	19 (95%)	1 (5%)	0 (0%)	0 (0%)	0 (0%)	0 (0%)	0 (0%)	0.798
	Symptomatic one RECI	20 (100%)	0 (0%)	0 (0%)	0 (0%)	0 (0%)	0 (0%)	0 (0%)	
	Asymptomatic one curve	18 (90%)	0 (0%)	0 (0%)	1 (5%)	1 (5%)	0 (0%)	0 (0%)	
	Symptomatic one curve	18 (90%)	1 (5%)	0 (0%)	1 (5%)	0 (0%)	0 (0%)	0 (0%)	

^*x*^Fisher–Freeman–Halton test.

**Table 5:** An analgesic intake on postoperative pain in each time interval.

Group	Postoperative time	Analgesic intake						*p* value^*x*^
		No intake			Intake			
		*n*	Mean ± SD	Median (range)	*n*	Mean ± SD	Median (range)	
Asymptomatic one RECI	24 h	14	1.02 ± 1.75	0 (5)	6	5.17 ± 2.04	5.5 (6)	< 0.001
	48 h	14	1.02 ± 1.75	0 (5)	6	5 ± 2.76	5 (6)	0.003
	72 h	14	0.5 ± 1.4	0 (5)	6	4.83 ± 4.12	5.5 (10)	0.033
	Day 7	14	0.07 ± 0.27	0 (1)	6	1.5 ± 1.97	1 (5)	0.109
	Day 14	14	0.07 ± 0.27	0 (1)	6	0.17 ± 0.41	0 (1)	0.779

Symptomatic one RECI	24 h	11	0.73 ± 1.35	0 (4)	9	5.78 ± 2.49	6 (8)	< 0.001
	48 h	11	0.64 ± 1.12	0 (3)	9	5.78 ± 2.44	6 (7)	< 0.001
	72 h	11	0.55 ± 0.93	0 (2)	9	4.89 ± 2.67	5 (8)	0.001
	Day 7	11	0.09 ± 0.3	0 (1)	9	1.22 ± 2.28	0 (7)	0.175
	Day 14	11	0 ± 0	0 (0)	9	0 ± 0	0 (0)	1

Asymptomatic one curve mini	24 h	16	0.88 ± 1.31	0 (4)	4	7 ± 2.94	7 (6)	< 0.001
	48 h	16	0.44 ± 0.81	0 (2)	4	6.5 ± 3.11	6.5 (7)	< 0.001
	72 h	16	0.19 ± 0.4	0 (1)	4	6 ± 3.16	6.5 (7)	< 0.001
	Day 7	16	0.06 ± 0.25	0 (1)	4	3.5 ± 3.11	3.5 (7)	0.022
	Day 14	16	0.06 ± 0.25	0 (1)	4	2.75 ± 3.2	2.5 (6)	0.178

Symptomatic one curve	24 h	16	1.13 ± 2.28	0 (8)	4	8.5 ± 1.29	8.5 (3)	0.001
	48 h	16	0.81 ± 1.94	0 (7)	4	7 ± 0.82	7 (2)	0.002
	72 h	16	0.75 ± 1.88	0 (7)	4	3.5 ± 2.52	4 (6)	0.064
	Day 7	16	0.56 ± 1.55	0 (6)	4	1 ± 2	0 (4)	0.82
	Day 14	16	0.37 ± 1.26	0 (5)	4	0.25 ± 0.5	0 (1)	0.75

Total	24 h	57	0.95 ± 1.71	0 (8)	23	6.3 ± 2.46	6 (9)	< 0.001
	48 h	57	0.72 ± 1.47	0 (7)	23	5.91 ± 2.41	6 (9)	< 0.001
	72 h	57	0.49 ± 1.28	0 (7)	23	4.83 ± 3.04	5 (10)	< 0.001
	Day 7	57	0.21 ± 0.86	0 (6)	23	1.65 ± 2.33	0 (7)	< 0.001
	Day 14	57	0.14 ± 0.69	0 (5)	23	0.57 ± 1.59	0 (6)	0.15

^*x*^Mann–Whitney *U* test.

## Data Availability

The data are not publicly available due to privacy or ethical restrictions. The data that support the findings of this study are available on request from the corresponding author (Hüseyin Gürkan Güneç).
